# Molecular Characterization of a 21.4 Kilobase Antibiotic Resistance Plasmid from an α-Hemolytic *Escherichia coli* O108:H- Human Clinical Isolate

**DOI:** 10.1371/journal.pone.0034718

**Published:** 2012-04-20

**Authors:** Fay E. Dawes, Dieter M. Bulach, Alexander Kuzevski, Karl A. Bettelheim, Carola Venturini, Steven P. Djordjevic, Mark J. Walker

**Affiliations:** 1 School of Biological Sciences, University of Wollongong, New South Wales, Australia; 2 Department of Microbiology and Victorian Bioinformatics Consortium, Monash University, Clayton, Victoria, Australia; 3 Microbiological Diagnostic Unit, Department of Microbiology and Immunology, University of Melbourne, Parkville, Victoria, Australia; 4 School of Chemistry and Molecular Biosciences and the Australian Infectious Diseases Research Centre, University of Queensland, Queensland, Brisbane, Australia; 5 Microbiology and Immunology Section, Industry and Investment NSW, Elizabeth Macarthur Agricultural Institute, Menangle, New South Wales, Australia; 6 The ithree Institute, University of Technology, Sydney, New South Wales, Australia; Naval Research Laboratory, United States of America

## Abstract

This study characterizes the 21.4 kilobase plasmid pECTm80 isolated from *Escherichia coli* strain 80, an α hemolytic human clinical diarrhoeal isolate (serotype O108:H-). DNA sequence analysis of pECTm80 revealed it belonged to incompatibility group X1, and contained plasmid partition and toxin-antitoxin systems, an R6K-like triple origin (*ori*) replication system, genes required for replication regulation, insertion sequences IS*1R,* IS*Ec37* and a truncated transposase gene (Tn*3-*like *ΔtnpA*) of the Tn*3* family, and carried a class 2 integron. The class 2 integron of pECTm80 contains an intact cassette array *dfrA1-sat2*, encoding resistance to trimethoprim and streptothricin, and an *aadA1* gene cassette truncated by the insertion of IS*1R*. The complex plasmid replication system includes α, β and γ origins of replication. Pairwise BLASTn comparison of pECTm80 with plasmid pE001 reveals a conserved plasmid backbone suggestive of a common ancestral lineage. Plasmid pECTm80 is of potential clinical importance, as it carries multiple genes to ensure its stable maintenance through successive bacterial cell divisions and multiple antibiotic resistance genes.

## Introduction

The dissemination of multi-drug resistant bacteria is a serious and growing global health threat. Infections caused by multi-drug resistant pathogens that fail to respond to treatment, often result in prolonged illness and an increased risk of death. Understanding the molecular mechanisms that facilitate the clustering and horizontal transfer of antibiotic resistance genes is essential to the development of strategies that address this growing problem in the treatment of infectious diseases.

Horizontal gene transfer of antibiotic resistance genes may occur via various genetic elements including transformation or conjugation of plasmids, mobilizable plasmids, conjugative transposons and phages [1], [2]. Plasmids belonging to the incompatibility (Inc) group X have been implicated in the acquisition and spread of antibiotic resistance-transposons such as Tn*7*, Tn*3* and Tn*21* in pathogenic enterobacteria [3], [4]. IS elements may facilitate the dissemination of resistance genes and participate in chromosomal and plasmid rearrangement, integration and excision [5]. Integrons play an important role in the emergence of multi-drug resistant pathogens by functioning as mobile gene cassette capture and expression systems.

Class 2 integron screening studies frequently identify the Tn*7*-type cassette array of *dfrA1-sat2-aadA1,* which confers resistance to trimethoprim, streptothricin, streptomycin and spectinomycin. Class 2 integrons are typically located at a unique site near the end of the non-replicative transposon Tn*7* or related transposons including Tn*1825,* Tn*1826* and Tn*4132*, which provide a means for their mobilization [6], [7]. This study characterizes an *Escherichia coli* isolate that harbors an antibiotic resistance plasmid containing a class 2 integron.

## Methods

### Strain isolation and characterization

The *Escherichia coli* isolate characterized in this study, designated strain 80, was recovered from a patient with clinical diarrhea and submitted to the Microbiological Diagnostic Unit (MDU, Public Health Laboratory, Department of Microbiology and Immunology, University of Melbourne, Victoria, Australia). The strain was identified as *E. coli* by culture in specialized media, whilst O- and H-serotyping was performed as previously described [8]. Carriage of virulence determinants by *E. coli* strain 80 was examined by detection of Shiga toxins [9], [10] and α-haemolysin [11]. Sensitivity to the following antibiotics was determined using the plate/replicator method as described by Bettelheim *et al*. [8]: ampicillin (32 µg ml^−1^), streptomycin (25 µg ml^−1^), tetracycline (20 µg ml^−1^), chloramphenicol (10 µg ml^−1^), sulfathiazole (550 µg ml^−1^) trimethoprim (50 µg ml^−1^), kanamycin (10 µg ml^−1^), nalidixic acid (50 µg ml^−1^), spectinomycin (50 µg ml^−1^), gentamicin (2.5 µg ml^−1^) and ciprofloxacin (2 µg ml^−1^).

### Class 2 integron detection

Class 2 integron carriage was detected by PCR screening for the *intI2* gene using primers [12] and cycling conditions [13] described previously. Confirmation of DNA integrity and strain identification as *E. coli* was achieved by PCR of the *E.coli*-specific universal stress protein A (*uspA*) gene. PCR amplification of the *uspA* gene was carried out simultaneously with amplification of the *intI2* gene using previously described primers EC2 [14] and FD-uspAF [13]. *E. coli* strain DH5α harboring the plasmid pMAQ612 (ampicillin^R^; *intI2* from Tn*7* cloned into pUC18) was used as the positive control for PCR [15]. Southern hybridization of plasmid DNA versus total genomic DNA was performed to establish the genomic location of the *intI2* gene. Genomic DNA was extracted using the DNeasy tissue kit (Qiagen) and digoxigenin (DIG)-labelled *intI2* PCR product amplified using the primers Int2.F and Int2.R [12] was used as a probe.

### Nucleotide sequence and annotation of pECTm80

Plasmid DNA isolated from *E. coli* strain 80 was transformed by electroporation into *E. coli* JM109 using standard methods [16] and the complete DNA sequence of the plasmid, designated pECTm80 was determined. DNA sequencing was performed according to the manufacturer's instructions using the BigDye Terminator v3.1 cycle sequencing kit (Perkin-Elmer) and the 3130 Genetic Analyzer capillary sequencer (Applied Biosystems). Both strands of the plasmid were sequenced by employing a primer walking strategy. To facilitate sequencing, *Hin*dIII digested plasmid fragments of 2.6 kb, 6.8 kb and 12.6 kb were cloned into pUC18 (Fermentas). Sequencing of pECTm80 proceeded preferentially from the original plasmid and from the recombinant plasmids containing pECTm80 inserts as required. Plasmid DNA was extracted using the plasmid maxi kit (Qiagen) or the Wizard Miniprep DNA Purification System (Promega). Oligonucleotide primers were designed with the Primer3 program [17] and synthesized by Sigma, Sydney, Australia.

Contig Express by Vector NTI Advance 10 (Invitrogen) was utilized to align contiguous sequences. The CDS (coding sequences) were identified using the gene finder program GeneMarkS [18]. Annotation of CDS was achieved by performing BLAST searches available from NCBI and IS BLAST server (http://www-is.biotoul.fr). Annotated sequences were visualized using Artemis (version 13.2) [19]. Comparative DNA analysis was performed by visualizing pairwise BLASTn comparisons with the Easyfig program available at http://easyfig.sourceforge.net/ [20].

### Plasmid conjugation assay

The ability to transfer resistance by conjugation was performed as previously described [21]. Matings were carried out with the transformant *E.coli* strain JM109 (pECTm80) and recipient *E. coli* strain 294 Rif^r^ Nal^r^ (rifampicin and nalidixic acid resistant; β-galactosidase positive) [22] with and without the addition of *E.coli* HB101 containing the conjugal helper plasmid pRK600 Cm^r^ (chloramphenicol resistant) [23].

**Figure 1:**
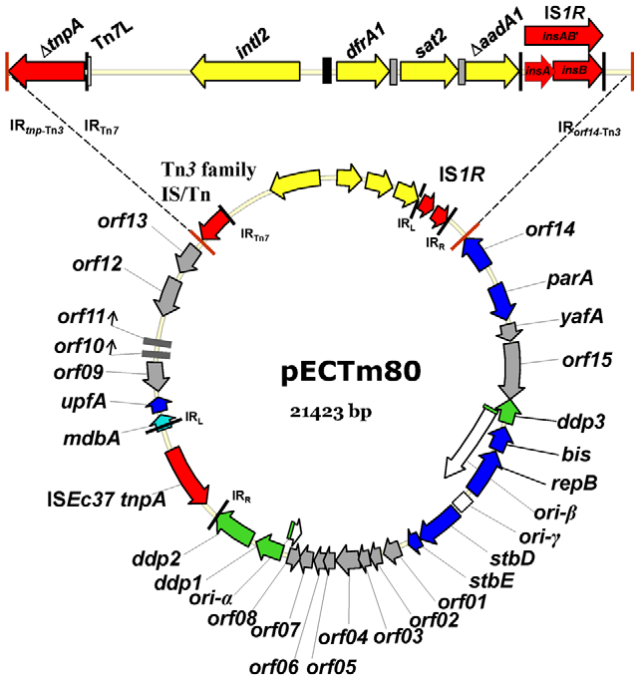
Illustration of plasmid pECTm80 isolated from *E. coli* strain 80 (GenBank no. FJ914220). Arrows indicate the direction of transcription. Terminal inverted repeats (IR) of transposons and insertion sequences (IS) (left IR, IR_L_ and right IR, IR_R_) are indicated: 38 bp IR of a Tn*3* family transposon/IS remnant adjacent to the Tn*3-*like Δ*tnpA* gene (IR*_tnp_*_-Tn*3*_) and *orf14* (IR*_orf14_*_-Tn*3*_) (red), 23 bp IR IS*1R* [43], 8 bp imperfect terminal IRs carried by IS*Ec37*, 8 bp IR at the left end of Tn*7* (IR_Tn*7*_). Tn*7*L denotes 150 bp at the left end of Tn*7* containing multiple TnsB binding sites (open box). The location of the three origins of replication are indicated (α and β, unfilled arrows; and γ, unfilled box). The arrows mark the *in vivo* direction of the initial replication from the α and β origins. The origins of conjugal transfer (*oriT*α and *oriT*β) are indicated by a green filled box. Colour-coded functional categories of predicted CDSs include insertion sequence/transposon transposases (red); conjugal transfer (*ddp1*, *ddp2* and *ddp3* genes: green); plasmid maintenance and stability including replication initiation (*bis* and *repB* genes), plasmid partitioning (*orf14* and *parA* genes) and plasmid stability (*stbD* gene: toxin and *stbE* gene: anti-toxin) (blue); gene expression modulation (*mdbA* gene: light blue); and hypothetical proteins of unknown function (grey). Integron features indicated include *intI2* and cassette genes (yellow arrows), *attI2* (closed box) and *attC* (grey boxes).

**Table 1:** Identification of CDS in the nucleotide sequence of pECTm80.

Gene/ORF	Strand	pECTm80 coordinates	Function/name of protein	Accession no.^A^	Plasmid^B^	% Identity
*ddp3 (taxD)*	−	3–425	DNA distortion protein 3- DNA transfer element	JF776874.1	1	100
*bis*	−	496–939	Replication initiation protein	JF776874.1	1	100
*repB (repX)*	−	967–1818	Iteron binding initiator of plasmid replication	JF776874.1	1, 2, 3	100
*stbD*	+	2800–3128	Stability protein StbD	JF776874.1	1, 2, 3, 4, 5, 6	100
*stbE*	+	3121–3399	Stability protein StbE	JF776874.1	1, 4	100
*orf01*	+	3548–3871	Conserved hypothetical protein	JF776874.1	1, 4	100
*orf02*	+	3904–4157	Conserved hypothetical protein	JF776874.1	1, 4	100
*orf03*	+	4145–4366	Conserved hypothetical protein	JF776874.1	1, 4	100
*orf04*	+	4360–4788	Conserved hypothetical protein	JF776874.1	1, 4	99
*orf05*	+	4831–5010	Conserved hypothetical protein	JF776874.1	1, 4	100
*orf06*	+	5032–5229	Conserved hypothetical protein	JF776874.1	1, 4, 5, 6	100
*orf07*	+	5242–5511	Conserved hypothetical protein	JF776874.1	1, 2, 4	100
*orf08*	_	5485–5789	Conserved hypothetical protein	JF776874.1	1	100
*ddp1 (taxA)*	+	5840–6382	DNA distortion protein 1-DNA transfer auxiliary protein	JF776874.1	1	100
*ddp2 (taxC)*	+	6382–7359	DNA distortion protein 2- DNA transfer relaxase	JF776874.1	1	100
IS*Ec37^C^ tnpA*	_	7545–8729	IS*Ec37* transposase	AE005674.2	NA	97
*mdbA*	+	9113–9385	Putative DNA-binding protein H-NS histone family	DQ115387.2	5, 6	100
*upfA*	+	9439–9699	UpfA conserved hypothetical protein	JF776874.1	1, 3	100
*orf09*	_	10269–10528	Conserved hypothetical protein	JF776874.1	1, 7	100
*orf10*	+	10458–10601	Conserved hypothetical protein SCH_083	JF776874.1	1	100
*orf11*	+	10671–10847	Conserved hypothetical protein	JF776874.1	1, 2, 3, 5, 6	100
*orf12*	_	10933–11751	Conserved hypothetical protein	JF776874.1	1	100
*orf13*	_	12001–12525	Conserved hypothetical protein	JF776874.1	1	100
Tn*3*-like^D^ Δ*tnpA* ^E^	_	12697–13368	Transposase truncated N-terminus by Tn*7*	EU330199	NA	100
*intI2*	_	14309–15283	Class 2 integrase	AB188272	NA	100
*dfrA1*	+	15615–16088	Dihydrofolate reductase	AB188272	NA	100
*sat2*	+	16183–16704	Streptothricin acetyltransferase	AB188272	NA	100
*ΔaadA1*	+	16765–17244	Aminoglycoside adenylyltransferase	AB188272	NA	100
IS*1R* ^F^ *insA*	+	17295–17569	InsA transcription repressor	CP002890.1	NA	98
IS*1R insB*	+	17549–17992	None	AE005674.2	NA	98
IS*1R insAB’*	+	17295–17548 17548-17992	IS*1R* transposase InsAB’(frame shift product)	CP002890.1	NA	98
*orf14*	_	18365–19018	Partitioning (par)-Resolvase	JF776874.1	1	100
*parA (yafB)*	+	19381–20040	Plasmid partition protein A	JF776874.1	1, 5	100
*yafA*	+	20116–20421	Conserved hypothetical protein YafA	JF776874.1	1	100
*orf15*	+	20449–21423	Conserved hypothetical protein	JF776874.1	1	100

AGenBank accession numbers provided represent the results of BLAST searches (NCBI and IS BLAST server) showing the highest identity to the query sequence. The accession number for pE001 is given when there is greater than one BLAST hit at 100% ID to pECTM80. BPlasmids showing 100% ID to pECTM80 are represented as follows: 1, pE001 (JF776874.1); 2, R485 (HE577112.1); 3, pMAS2027 (FJ666132.1); 4, pOLA52 (EU370913.1); 5, pOU1114 (DQ115387.2); 6, pSE34 (EU219533.1); and 7, pMccC7-H22 (EF536825.1). Boundaries of mobile elements in the nucleotide sequence of pECTm80 are as follows: CISEc37, 7355–9182; DTn3-like, 12671–18314; FIS1R, 17240–18007. ETn3-like ΔtnpA gene showed 64% to 76% identity to transposase genes of the Tn3 family of transposons (subgroups Tn501 and Tn3) and the IS elements ISSba14, and ISSod9 of the Tn3 family.

## Results

O- and H-serotyping revealed that *E. coli* strain 80 possessed the serotype O108:H-. Strain 80 was found to produce α-hemolysin but did not produce either of the Shiga toxins. *E. coli* strain 80 displayed resistance to multiple antibiotics including streptomycin (25 µg ml^−1^), tetracycline (20 µg ml^−1^), sulfathiazole (550 µg ml^−1^), trimethoprim (50 µg ml^−1^) and nalidixic acid (50 µg ml^−1^). PCR detection of both *uspA* and *intI2* genes confirmed strain 80 was an *E. coli* isolate and contained a class 2 integron. Southern blot analysis of plasmid versus total genomic DNA using a DIG-labeled probe revealed the *intI2* gene was located on a plasmid. The mating-out assay revealed the plasmid from *E. coli* strain 80 was not conjugative and was not able to be mobilized by pRK600 (results not shown).

The annotated DNA sequence of plasmid pECTm80 is deposited in GenBank (FJ914220) and described in Fig. 1 and Table 1. DNA sequence analysis revealed significant features of the plasmid sequence including plasmid partition and toxin-antitoxin systems, a complex replication system, genes required for conjugative transfer and replication regulation, a truncated Tn*7* class 2 integron, insertion sequences and a truncated Tn*3* family IS element or transposon remnant. An IS*1* isoform that showed 98% nucleotide identity to IS*1R* (GenBank J01730) and a new IS element IS*Ec37* belonging to the IS*91* family were identified in pECTm80. IS*Ec37* is 95% identical to IS*91* (GenBank X17114) and the encoded transposases show 95% amino acid sequence identity.

An IS*1R*-mediated deletion is observed in the Tn*7-*like class 2 integron. This class 2 integron contains intact gene cassettes *dfrA1* and *sat2* encoding resistance to trimethoprim and streptothricin, and an *aadA1* cassette gene (Δ*aadA1*) truncated by the insertion of IS*1R*. The open reading frames (ORFs) *ybeA* (*orfX*), *ybfA, ybfB,* and Tn*7* transposition genes *tnsABCDE*, which are usually found at the 3′ end of class 2 integrons were also deleted by IS*1R* insertion. Consistent with other class 2 integrons, the integrase gene *intI2* contains a premature in-frame stop codon, which typically encodes a defective IntI2 protein [24].

A Tn*3* family transposase gene (Tn*3*-like Δ*tnpA*), located at the 5′ end of the class 2 integron, contains a Tn*7*-mediated truncation. An 8 bp IR at the left end of Tn*7* (IR_Tn*7*_) is adjacent to the Tn*3*-like Δ*tnpA* deletion site. The complete ΔTn*3*-like Δ*tnpA*-*Δ*Tn*7* class 2 integron-IS*1R* gene configuration is flanked by Tn*3* 38 bp IRs (IR*_tnp_*_-Tn*3*_ and IR*_orf14_*_-Tn*3*_). The 5 bp direct repeats (TATAT) characteristic of Tn*3* insertion are located adjacent to the Tn*3* 38 bp IRs. BLAST results (NCBI and IS BLAST server), indicate Tn*3*-like *ΔtnpA* is a remnant of a transposon or IS element of the Tn*3* family. Tn*3*-like *ΔtnpA* showed 64–76% identity to transposase genes of transposons belonging to the Tn*3* family (subgroups Tn*501* and Tn*3*; GenBank: X90708.2 and Y00502.1) and IS elements IS*Sba14*, and IS*Sod9* (IS Finder; GenBank NC_009052.1 and NC_004349) also of the Tn*3* family. The highest identity was displayed to IS*Sba14* and Tn*2501* (76% identity and 87% query coverage).

The replication system identified in pECTm80 consists of an origin (*ori*) of plasmid replication region that spans 7.6 kb. This region contains α, β and γ origins of replication, three DNA distortion protein genes (*ddp1*, *ddp2* and *ddp3*) encoding proteins involved in conjugative transfer and the regulation of replication [25], [26], and the initiation transfer genes *bis* and *repB* [27]. *oriT*α and *oriT*β were identified within the α and β long inverted repeat nucleotide sequences of pECTm80.

**Figure 2:**
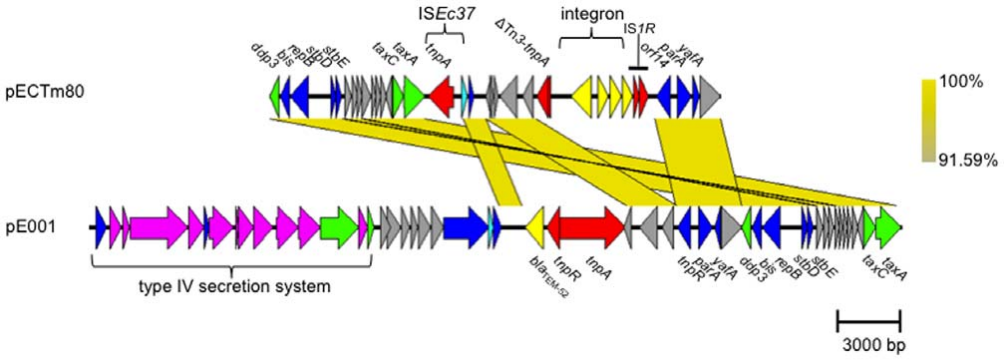
Comparison of pECTm80 with the *E. coli* plasmid pE001. Pairwise BLASTn comparisons between the plasmids pECTm80 and pE001 were visualized using the Easyfig program [23]. Regions of nucleotide identity are connected by yellow blocks. The yellow color gradient indicates the extent of similarity as shown in the color scale on the right. Functions of CDS in pE001 have been taken from BLAST matches and existing annotation. Functional categories of predicted CDSs include: insertion sequence/transposon transposases (red); conjugal transfer (green); plasmid maintenance and stability (blue); gene expression modulation (light blue); class 2 integron *intI2* gene and gene cassettes (yellow); type IV secretion system (mauve); bla_TEM-52_ (yellow) and hypothetical proteins of unknown function (grey). Scale bar represents 3000 base pairs.

Nucleotide sequence of pECTm80 showed 99–100% identity (65% pECTm80 coverage) with a 38.6 kb IncX1 plasmid, pE001, isolated from *E. coli* strain 2161 in broiler meat in Denmark (GenBank JF776874.1) [28]. A diagram of the pairwise BLASTn comparison between plasmids pECTm80 and pE001 is given in Figure 2. pECTm80 also showed 90–98% identity to the 34.5 kb plasmid pOU1114 (GenBank NC_010421) isolated from *Salmonella enterica* serovar Dublin (49% pECTm80 coverage and 18 CDS) [29] and 89–99% identity to the 51.6 kb plasmid pOLA52 (GenBank EU370913) isolated from *Escherichia coli* (39% pECTm80 coverage and 14 CDS) [30]. The plasmids pECTm80 and pE001 shared a common backbone showing 99.9% nucleotide sequence identity. Plasmid pE001 did not contain Tn*3*-like Δ*tnpA,* a class 2 integron, IS*Ec37* nor IS*1R* found in pECTm80 while pECTm80 did not contain the *pilX* operon, found on pE001 and other conjugative IncX1 plasmids [28].

## Discussion

This study characterizes pECTm80, a 21.4 kilobase IncX1 plasmid containing a truncated class 2 integron from an *Escherichia coli* O108:H- human clinical isolate. *E.coli* strain 80 serogroup O108 was non–motile (H-) and produced α-hemolysin, a potent enterotoxin that is known to enhance virulence in a number of clinical infections [31], although strain 80 did not produce either of the Shiga toxins. DNA sequence analysis of pECTm80 revealed a Tn*7* class 2 integron with complete *dfrA1* and *sat2* gene cassettes and an IS*1R-*mediated deletion resulting in truncation of the *aadA1* gene cassette and deletion of the ORFs *ybeA* (*orfX*), *ybfA* and *ybfB,* and Tn*7* transposition genes *tnsABCDE*.

Several key features of the integron-containing plasmid pECTm80 include the presence of plasmid partitioning and segregational stability genes to ensure the stable maintenance of the plasmid through successive bacterial cell divisions and a highly–regulated DNA replication system consisting of three distinct origins of replication α, β and γ, that are controlled and activated by plasmid– and host-encoded genes [32]. Plasmid pECTm80 contains plasmid partitioning genes and toxin-antitoxin system genes, which ensure its vertical transfer. The plasmid partitioning protein ParA is encoded by the *parA (yafB)* gene and a partitioning (par)-resolvase by *orf14* [33]. The toxin-antitoxin system, encoded by *stbD* and *stbE* genes, promotes segregational stability by compromising the survival of plasmid-free daughter cells that may arise during cell division [34], [35], [36]. The complex replication system of pECTm80 is a strategy for increasing plasmid mobility to a range of hosts [37]. A study characterizing the host–range of an IncX plasmid of R6K lineage found the plasmid was established in 9 of 16 species tested [38]. Plasmid pECTm80 belongs to the IncX1 group, showing 96% identity to the IncX1 plasmid R485 replication origin region (GenBank M11688.1). Division of the IncX group into IncX1 and IncX2 arose following discovery of *in vivo* incompatibility between the IncX plasmids R485 and R6K, and lack of extensive similarity over long nucleotide stretches in these replicons [35]. R6K is the sole well-characterized member of the IncX2 group [39], although a second IncX2 plasmid was recently identified [40].

Nucleotide sequence analysis of pECTm80 also revealed this plasmid carries genes and sites necessary for plasmid mobilization including *ddp1, ddp2, ddp3, bis* and *repB* genes, and α and β oriTs. However pECTm80 was neither conjugative nor mobilized by the helper plasmid pRK600. pECTm80 did not contain genes of the *pilX* operon that encode a Type IV secretion system essential for mate-pair formation and conjugal transfer, which are found on conjugative IncX1 plasmids including pE001, pOLA52 and pOU1114 [29], [30], [41]. The absence of *pilX* genes may explain the non-conjugative properties of pECTm80. The deletion of the *pilX* operon may have resulted due to insertion of the IS*Ec37* element into pECTm80.

Close association of the Tn*7* class 2 integron with other mobile DNA elements including insertion sequences and transposons is reported in this study. The complex and dynamic interaction of mobilizable elements described here, act to increase their potential for gene shuffling [42]. The IS*1R-*mediated truncation of the Tn*7* class 2 integron and the IS*1R*- or Tn*7-*mediated truncation of the Tn*3-*like Δ*tnpA* are observed. The mosaic Tn*3*-like Δ*tnpA*-Δ**Tn*7* class 2 integron-IS*1R* gene configuration is flanked by Tn*3* 38 bp IRs (IR*_tnp_*_-Tn*3*_ and IR*_orf14_*_–Tn*3*_) and 5 bp target-site direct repeats (TATAT). The presence of IS*1R* and a Tn*3* family transposon/IS remnant with the class 2 integron implicates these DNA elements in the evolution of this unique integron. Sequential addition of Tn*3*-like (IS/transposon), Tn*7* and IS*1R* and the resulting insertion-mediated deletions are hypothesized to have occurred in pECTm80. The sequential addition of Tn*3* and Tn*7* to IncX1 plasmids leading to the creation of complex R-plasmids has been previously described [39].

Comparative DNA sequence analysis of pECTm80 with the conjugative IncX1 plasmid pE001, also isolated from an *E. coli* strain, provides insight into how this plasmid and its resident class 2 integron evolved. pECTm80 displayed a conserved backbone with archetypical IncX1 regions for replication and plasmid stability found in pE001 [29]. The shared essential regions for replication and plasmid stability and the high-level of identity described support the hypothesis that these plasmids have arisen from a common ancestral plasmid. The absence of IS*Ec37*, IS*1R*, the Tn*3* family transposon/IS remnant, and the class 2 integron in pE001 may suggest the integration of these elements into a common ancestral plasmid occurred to create pECTm80. Insertion sequences IS*Ec37* and IS*1R* are intact in pECTm80 and may represent recent insertions into the plasmid. Although the exact mechanism of evolution of the IncX1 plasmid pECTm80 and its resident class 2 integron remains unknown, multiple insertion, deletion and rearrangement events are likely to have occurred, as suggested by the proximity of several IS elements.

## References

[1] Normark HB, Normark S (2002). Evolution and spread of antibiotic resistance.. J Intern Med.

[2] Salyers A, Amabile-Cuevas C (1997). Why are antibiotic resistance genes so resistant to elimination?. Antimicrob Agents Chemother.

[3] Jones CS, Osborne DJ, Stanley J (1993). Molecular comparison of the IncX plasmids allows division into IncXl and IncX2 subgroups.. J Gen Microbiol.

[4] Threlfall EJ, Ward LR, Rowe B (1986). R plasmids in *Salmonella typhimurium* in the United Kingdom J Antimicrob Chemother.

[5] Mahillon J, Chandler M (1998). Insertion sequences.. Microbiol Mol Biol Rev.

[6] Sundström L, Roy PH, Sköld O (1991). Site-specific insertion of three structural gene cassettes in transposon Tn*7*.. J Bacteriol.

[7] Young H-K, Qumsieh MJ, McIntosh ML (1994). Nucleotide sequence and genetic analysis of the type Ib trimethoprim-resistant, Tn*4132*-encoded dihydrofolate reductase.. J Antimicrob Chemother.

[8] Bettelheim KA, Hornitzky MA, Djordjevic SP, Kuzevski A (2003). Antibiotic resistance among verocytotoxigenic *Escherichia coli* (VTEC) and non-VTEC isolated from domestic animals and humans.. J Med Microbiol.

[9] Konowalchuk J, Speirs JI, Stavric S (1977). Vero response to a cytotoxin of *Escherichia coli*.. Infect Immun.

[10] Acheson DW, Keusch GT, Lightowlers M, Donohue-Rolfe A (1990). Enzyme-linked immunosorbent assay for Shiga toxin and Shiga-like toxin II using P1 glycoprotein from hydatid cysts.. J Infect Dis.

[11] Bettelheim KA (1995). Identification of enterohaemorrhagic *Escherichia coli* by means of their production of enterohaemolysin.. J Appl Bacteriol.

[12] Mazel D, Dychinco B, Webb VA, Davies J (2000). Antibiotic resistance in the ECOR collection: integrons and identification of a novel *aad* gene.. Antimicrob Agents Chemother.

[13] Dawes FE, Kuzevski A, Bettelheim KA, Hornitzky MA, Djordjevic SP (2010). Distribution of class 1 integrons with IS*26*-mediated deletions in their 3'-Conserved Segments in *Escherichia coli* of human and animal origin.. PLoS ONE.

[14] Chen J, Griffiths MW (1998). PCR differentiation of *Escherichia coli* from other gram-negative bacteria using primers derived from the nucleotide sequences flanking the gene encoding the universal stress protein.. Lett Appl Microbiol.

[15] Dillon B, Thomas L, Mohmand G, Zelynski A, Iredell J (2005). Multiplex PCR for screening of integrons in bacterial lysates.. J Microbiol Methods 62: 221–.

[16] Sambrook J, Russell DW (2001). Molecular cloning: A laboratory manual..

[17] Rozen S, Skaletsky HJ (2000). Primer3 on the WWW for general users and for biologist programmers. In: Krawetz S, Misener S, editors. Bioinformatics methods and protocols: methods in molecular biology.. Totowa, NJ: Humana.

[18] Besemer J, Lomsadze A, Borodovsky M (2001). GeneMarkS: a self-training method for prediction of gene starts in microbial genomes. Implications for finding sequence motifs in regulatory regions.. Nucleic Acids Res.

[19] Rutherford K, Parkhill J, Crook J, Horsnell T, Rice P (2000). Artemis: sequence visualization and annotation.. Bioinformatics.

[20] Sullivan MJ, Petty NK, Beatson SA (2011). Easyfig: a genome comparison visualizer.. Bioinformatics.

[21] Venturini C, Beatson SA, Djordjevic SP, Walker MJ (2009). Multiple antibiotic resistance gene recruitment onto the enterohemorrhagic *Escherichia coli* virulence plasmid.. FASEB J.

[22] Talmadge K, Gilbert W (1980). Construction of plasmid vectors with unique *Pst*I cloning sites in a signal sequence coding region.. Gene 12.

[23] Finan TM, Kunkel B, De Vos GF, Signer ER (1986). Second symbiotic megaplasmid in *Rhizobium meliloti* carrying exopolysaccharide and thiamine synthesis genes.. J Bacteriol.

[24] Hansson K, Sundström L, Pelletier A, Roy PH (2002). IntI2 integron integrase in Tn*7*.. J Bacteriol.

[25] Núñez B, Avila P, De La Cruz F (1997). Genes involved in conjugative DNA processing of plasmid R6K.. Mol Microbiol.

[26] Flashner Y, Schlomai J, Shafferman A (1996). Three novel plasmid R6K proteins act in concert to distort DNA within the alpha and beta origins of DNA replication.. Mol Microbiol.

[27] Flashner Y, Shlomai J, Shafferman A (1996). Three novel plasmid R6K proteins act in concert to distort DNA within the α and β origins of DNA replication.. Mol Microbiol.

[28] Bielak E, Bergenholtz RD, Jørgensen MS, Sørensen SJ, Hansen LH (2011). Investigation of diversity of plasmids carrying the bla_TEM-52_ gene.. Journal of Antimicrobial Chemotherapy.

[29] Chu C, Chiu C-H (2006). Evolution of virulence plasmids of non-typhoid *Salmonella* and its association with antimicrobial resistance.. Microbes Infect.

[30] Norman A, Hansen LH, She Q, Sørensen SJ (2008). Nucleotide sequence of pOLA52: A conjugative IncX1 plasmid from *Escherichia coli* which enables biofilm formation and multidrug efflux.. Plasmid.

[31] Ørskov F (1978). Virulence factors of the bacterial cell surface.. J Infect Dis.

[32] Bidyut KM, Marc L, Deepak B, Pillarisetty VALR (1996). The Replication Initiator Protein Pi; of the Plasmid R6K Specifically Interacts with the Host-Encoded Helicase DnaB.. Proc Natl Acad Sci U S A.

[33] Abeles AL, Friedman SA, Austin SJ (1985). Partition of unit-copy miniplasmids to daughter cells. III. The DNA sequence and functional organization of the P1 partition region.. J Mol Biol.

[34] Jensen R, Gerdes K (1995). Programmed cell death in bacteria: proteic plasmid stabilization systems.. Mol Microbiol.

[35] Hayes F (1998). A family of stability determinants in pathogenic bacteria.. J Bacteriol.

[36] Holcik M, Iyer VM (1997). Conditionally lethal genes associated with bacterial plasmids.. Microbiology.

[37] Toukdarian A, Funnell B, Phillips G (2004). Plasmid strategies for broad-host-range replication in Gram-negative bacteria.. Plasmid Biology.

[38] Wild J, Czyz A, Rakowski SA, Filutowicz M (2004). γ Origin plasmids of R6K lineage replicate in diverse genera of Gram-negative bacteria.. Annal Microbiol.

[39] Jones CS, Osborne DJ, Stanley J (1993). Molecular comparison of the IncX plasmids allows division into IncX1 and IncX2 subgroups.. J Gen Microbiol.

[40] Literak I, Dolejska M, Janoszowska D, Hrusakova J, Meissner W (2010). Antibiotic-resistant *Escherichia coli* bacteria, including strains with genes encoding the extended-spectrum beta-lactamase and QnrS, in waterbirds on the Baltic Sea Coast of Poland.. Appl Environ Microbiol.

[41] Bielak E, Bergenholtz RD, Jorgensen MS, Sorensen SJ, Hansen LH (2010). Investigation of diversity of plasmids carrying the blaTEM-52 gene.. J Antimicrobial Chemother.

[42] Toussaint A, Merlin C (2002). Mobile elements as a combination of functional modules.. Plasmid.

[43] Ohtsubo H, Ohtsubo E (1978). Nucleotide sequence of an insertion element, IS*1*.. Proc Natl Acad Sci U S A.

